# Solar Radiation Drives the Plant Species Distribution in Urban Built-Up Areas

**DOI:** 10.3390/plants14040539

**Published:** 2025-02-10

**Authors:** Heyi Wei, Bo Huang, Mingshu Wang, Xuejun Liu

**Affiliations:** 1 Geodesign Research Centre, Jiangxi Normal University, Nanchang 330022, China; 2 Department of Geography and Resource Management, Chinese University of Hong Kong, Hong Kong, China; 3 School of Geographical & Earth Sciences, University of Glasgow, Glasgow G12 8QQ, UK; mingshu.wang@glasgow.ac.uk; 4 Department of Geography, University of Hong Kong, Hong Kong, China; bohuang@hku.hk; 5 School of Urban Design, Wuhan University, Wuhan 430072, China; xjliu@whu.edu.cn

**Keywords:** solar radiation, geographic information systems (GISs), spatial distribution, species richness, species diversity, urban ecology

## Abstract

Urban areas serve as critical habitats for numerous plant species. Existing studies suggest that, due to human-mediated introductions, urban environments often harbor a greater variety of plant species compared to suburban areas, potentially becoming focal points for biodiversity. Consequently, investigating the driving forces and complex mechanisms by which urban environmental factors influence plant species distribution is essential for establishing the theoretical foundation for urban biodiversity conservation and future urban planning and management. Solar radiation, among these factors, is a critical determinant of plant growth, development, and reproduction. However, there is a notable lack of research on how this factor affects the distribution of urban plant species and influences species’ richness and composition within plant communities. We present for the first time an analysis of how solar radiation drives the spatial distribution of plant species within the built-up areas of Nanchang City, China. Based on three years of monitoring and survey data from experimental sites, this study employs three evaluation models—Species Richness Index (R), Simpson’s Diversity Index (D), and Shannon–Wiener Index (H)—to analyze and validate the survey results. Additionally, MATLAB and ArcGIS Pro software are utilized for the numerical simulation and visualization of spatial data. Our study shows that areas with low solar radiation exhibit higher plant species richness, while plots with high plant diversity are primarily concentrated in regions with strong solar radiation. Moreover, the Diversity Index D proves to be more sensitive than the Shannon–Wiener Index (H) in evaluating the spatial distribution of plant species, making it a more suitable metric for studying urban plant diversity in our study area. Among the 18 plant species analyzed, Mulberry and Dandelion are predominantly dispersed by birds and wind, showing no significant correlation with solar radiation. This finding indicates that the spatial distribution of urban plant species is influenced by multiple interacting factors beyond solar radiation, highlighting the critical need for long-term observation, monitoring, and analysis. This study also suggests that shaded urban areas may serve as hubs of high species richness, while regions with relatively strong solar radiation can sustain greater plant diversity. These findings underscore the practical significance of this research, offering essential insights to guide urban planning and management strategies. Additionally, this study offers valuable insights for the future predictions of plant species distribution and potential areas of high plant diversity in various urban settings by integrating computational models, building data, Digital Elevation Models (DEMs), and land cover data.

## 1. Introduction

Extensive research has long demonstrated that anthropogenic urbanization is a primary driver of native plant species loss, leading to substantial reductions or shifts in plant diversity [1,2,3,4,5,6]. However, the number of species in urban environments has increased in some cases, primarily due to the proliferation of certain alien plant species [7,8]. Urbanization is projected to continue and intensify, particularly in developing and less developed regions, reaching its peak by 2050 [9]. This indicates that the urban built environment will persist as a significant factor influencing plant species habitats and their diversity in the future [10,11,12]. Indeed, given the inevitability of urbanization, investigating the impact of urban elements on the loss, introduction, and diversity dynamics of plant species holds significant value for the conservation and management of urban biodiversity [13,14,15,16,17,18].

In response to the IUCN’s initiative to protect species and their diversity, ecologists, urban planners, and landscape architects argue that metropolitan areas should develop strategic plans and frameworks to manage future urban development in a way that is more conducive to the preservation of urban species and their biodiversity [12,19,20,21,22]. As mentioned earlier, human factors, as the principal drivers of changes in plant species and their diversity in urban environments, have been extensively documented [23,24]. For example, urban construction activities have removed native vegetation cover, while urban greening has introduced exotic landscape plants. However, the secondary physical changes associated with the urbanization process, and their role as driving factors influencing plant species and biodiversity, remain underexplored. In cities, buildings, terrain, roads, and other artificial structures alter the spatiotemporal distribution of ecological elements, leading to spatial heterogeneity in factors such as solar radiation, moisture, and temperature. As a result, these factors shape the spatial distribution patterns of plant species in urban environments over the long term.

From another perspective, plant species possess inherent genetic traits, such as their requirements for moisture, temperature, and light. These characteristics dictate the ecological conditions in which they can survive and thrive, thereby influencing their ecological distribution [25,26,27,28]. Therefore, we can explore these patterns to predict the spatial distribution, growth levels, and diversity of urban plant species, and use this information to inform urban planning and ecological conservation. However, there are still several challenges to overcome in testing this. Firstly, finding standardized experimental sites in built-up areas is difficult due to the complexity of the urban environment and the influence of human activity.

When a specific resource factor in an environment becomes scarce, the ecological tolerance of plants to that factor becomes the key determinant of species distribution and growth, as well as the core factor influencing plant diversity. Studies have demonstrated that in the Loess Plateau region of northwest China, soil moisture and atmospheric humidity play a decisive role in shaping ecosystem plant diversity and primary productivity. Plant diversity exhibits a strong correlation with shallow soil water content, while higher humidity conditions are more conducive to maintaining diversity [29]. Further research has revealed that topography, by indirectly affecting soil moisture, plays a critical regulatory role in determining species composition and grassland plant diversity, with topographic wetness identified as a significant determinant of these ecological characteristics [30]. Similar studies have also highlighted the pivotal roles of soil depth and moisture status in shaping grassland plant richness [31]. Collectively, these findings underscore that under stress conditions, plant distribution and diversity are often governed by the limiting ecological factor, exemplifying the principle articulated in Liebig’s Law of the Minimum [32,33].

As highlighted in the literature [34], to explore the socioeconomically driven urban plant diversity in the Central Arizona–Phoenix region, USA, experimental designs must incorporate variables such as land use and geospatial location. The intensity of urban landscape modification significantly impacts the spatial patterns of plant species richness [11]. Moreover, methods for assessing urban ecological environments using instruments and software remain underdeveloped, particularly in terms of continuous monitoring and simulation for specific areas. In recent years, advancements in computing and digital technologies have offered potential solutions to these challenges, including CFD-based urban wind modeling [35], urban thermal radiation simulations [36], and solar radiation simulation techniques [37,38,39]. Regarding the focus of this study—solar radiation as an environmental factor—our recent work has utilized digital technologies to enable the prediction of sunlight requirements for landscape plants [40], complemented by earlier related applications [41,42].

In urban built environments, solar radiation is considered a critical environmental factor influencing the distribution and composition of plant communities. However, existing research presents conflicting findings regarding its role. For instance, a study conducted on rooftop experimental sites, where solar radiation intensity was artificially manipulated, found that solar radiation had no significant effect on absolute vegetation cover. However, species composition varied significantly under different radiation intensities [43]. Another study, focused on the central region of Tibet, China, analyzed changes in plant species richness, evenness, and composition along an environmental gradient [44]. The results indicated that species composition was strongly associated with environmental variables such as soil moisture and elevation, but solar radiation showed no significant influence. Moreover, some studies have highlighted human activities, such as nitrogen deposition and grazing intensity, as major drivers of biodiversity loss in European grasslands [45]. In contrast, research conducted in suburban landscapes of Kladno, Czech Republic, identified land cover characteristics, including slope, solar radiation, bedrock, and landscape structure, as critical determinants of species diversity and composition [46]. While these studies illustrate the potential influence of solar radiation under specific environmental conditions, the role of solar radiation as a driving force for the spatial distribution of plant species in highly urbanized built environments remains underexplored.

To address this knowledge gap, this study aims to explore whether solar radiation can influence the spatial distribution of plant species in urban areas and to assess the sensitivity of various plant species to solar radiation in Nanchang, China. Field surveys recorded plant species, richness, abundance, and growth levels. Simultaneously, solar radiation across the study area was simulated using digital software and further calibrated with solar radiation data from mobile weather stations.

The specific objectives of this study are as follows: (1) to examine whether plant richness and diversity are affected by the intensity of solar radiation, and (2) to analyze the impact of solar radiation on plant abundance and growth dynamics.

By achieving these objectives, this research seeks to address the current gaps in understanding the influence of solar radiation on plant distribution in urban environments. Furthermore, it aims to provide valuable data support for urban planning and management by proposing a reliable predictive framework based on plant light-requirement niches and ecological amplitude theories. This framework will help identify potential urban plant diversity hotspots and spatial distribution patterns. Combining long-term monitoring with advanced digital simulation, this innovative approach offers new perspectives for theoretical research and practical solutions for sustainable urban development.

## 2. Results and Discussion

### 2.1. The Role of Solar Radiation in Shaping Species Richness and Diversity

**Figure 1:**
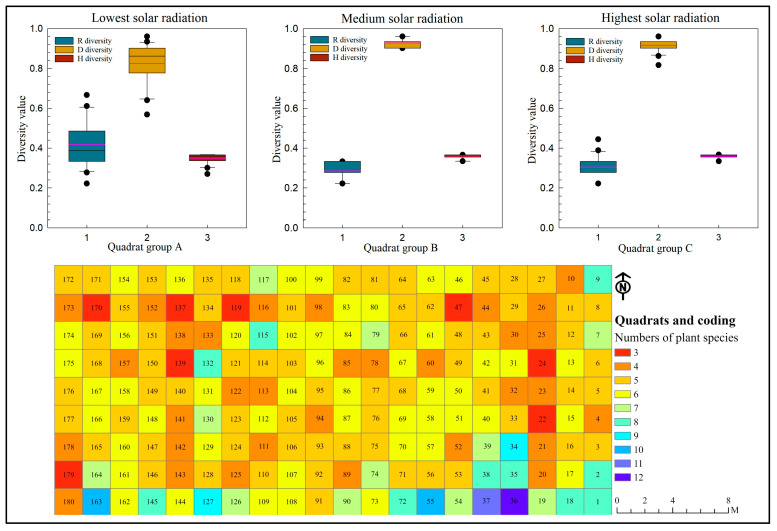
Distribution of plant richness and diversity driven by solar radiation.

Although sunlight is an essential factor for plant photosynthesis, its impact on plant diversity and species richness is complex. Our results indicate that, for the Species Richness Index R, the mean values are ranked in the following order: Group A > Group C > Group B (Figure 1), suggesting that the average species richness is higher in areas with lower solar radiation. Moreover, the dispersion and variability of the R index exhibit a similar trend, indicating that plant species richness varies more widely in areas with both low and high solar radiation. In contrast, habitats with medium levels of solar radiation tend to have lower species richness, with more uniform values and smaller variability, especially when compared to regions with lower solar radiation.

However, significant outliers were observed in certain sample plots in both high and low solar radiation areas. Reverse data validation revealed that these outliers were primarily concentrated in sample plots 9, 10, 36, and 180. Further analysis suggests that these plots are mostly located at the edges of buildings or roads, representing transitional zones between different habitats. In these areas, plant communities exhibit what is known in ecological theory as the edge effect, where species numbers show extreme increases or decreases. Therefore, this phenomenon warrants further in-depth investigation and validation, particularly with regard to the variability in soil moisture and growth space observed in edge areas.

Recent studies have investigated how geodiversity influences species richness and biodiversity [47,48,49]. The heterogeneity of the physical environment plays a significant role in shaping plant diversity [50], with evidence suggesting that geodiversity enhances vegetation biodiversity and positively affects plant community structure and species richness, particularly in drier years. Solar radiation, as a key physical factor for plants, means that its variation has the potential to support species diversity. This concept is further supported by niche theory and its applications [51,52,53,54,55].

Species richness and diversity are two distinct concepts with different mathematical implications. In this study, the diversity indices D (Simpson’s Index) and H (Shannon–Wiener Index) exhibit significant differences compared to the Species Richness Index R. As shown in Figure 1, the mean values of the Diversity Index D for sample plots with varying levels of solar radiation are ranked as follows: Group C > Group B > Group A. Additionally, the variability of the D index is significantly higher in Group A than in Groups B and C, indicating that shaded areas tend to result in the uneven distribution of plant species, as reflected by the D index. In contrast, the variability of the H index among Groups A, B, and C shows less differentiation, although it follows a similar trend. This comparison suggests that the D index is more sensitive than the H index in capturing urban plant diversity, as it better highlights the differences. On the other hand, the Species Richness Index R provides a more direct representation of the number of species in the sample plots. However, further validation across different geographical scales and for other taxonomic groups (such as animals, insects, and plants) is necessary to confirm these findings.

Our research data (Figure 1) indicate that the number of plant species is generally higher in areas near buildings. For instance, quadrats No. 36, 37, 55, and 163 each contain 10 or more species. This phenomenon is likely influenced by vortex winds generated in the built environment, which may facilitate the establishment and distribution of wind-dispersed plants. However, this hypothesis requires further evidence for validation. Additionally, through observation and tracking in the study area, we identified bird-dispersed species, such as *Mulberry*, which are spread via bird droppings. A detailed analysis of this bird-mediated dispersal mechanism is presented in Section 3.4.

For annual and biennial plants, the current species distribution remains unstable, indicative of a non-climax community, despite undergoing several life cycles since the study area’s protection. The emergence of trees and shrubs is expected to further alter the light environment [40,41,42,56], which, in turn, will drive subsequent changes in the spatial distribution patterns of plant species [57]. In habitats with varying levels of solar radiation, the eventual distribution of plant species requires long-term monitoring and further investigation. Understanding how environmental factors influence the spatial distribution of urban plant species is fundamental to urban biodiversity conservation and management [58,59,60,61,62]. Future research should focus on providing more comprehensive evidence on how built-up areas shape species distribution and community composition over time.

### 2.2. The Influence of Solar Radiation on Species Abundance

**Figure 2:**
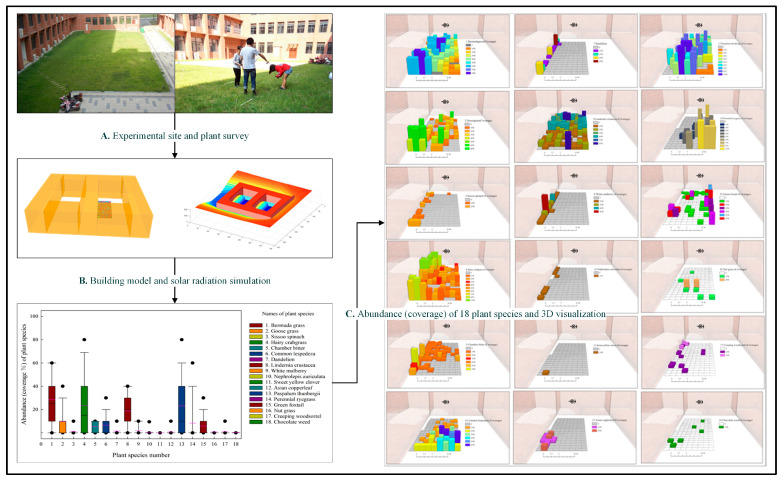
Abundance (coverage) of plant species in quadrats under different solar radiation levels.

*Bermudagrass*, initially sown as the primary grass species, has been influenced by natural forces such as self-adaptation and species competition over three years, following the site’s protection. During this period, the species abundance within the plots showed significant variation (Figure 2 and Figure S1 in Supplementary Material). Our findings indicate that urban lawns, particularly those dominated by Bermudagrass, are unsustainable without regular maintenance. This observation aligns with ongoing discussions in academia [63,64,65], which emphasize the need for urban green infrastructure planning and management to adopt nature-based solutions. Such approaches not only enhance ecological resilience but also reduce carbon emissions, contributing to urban sustainability. Among the exotic wild grasses observed, *Hairy Crabgrass*, *Lindernia crustacea*, and *Paspalum thunbergia* exhibited abundances comparable to that of *Bermudagrass* and are likely to spread further in the future. However, for these three species, no significant correlation was found between species abundance and solar radiation intensity.

In quadrats with lower solar radiation, species such as *Sissoo spinach*, *Chamber bitter*, *Dandelion*, *Mulberry*, and *Nephrolepis auriculata* exhibit higher coverage. A significant negative correlation was observed between species abundance and solar radiation intensity. However, for bird-dispersed and wind-dispersed plants like *Mulberry* and *Dandelion*, further long-term tracking and verification are required to confirm this conclusion. *Nephrolepis auriculata*, a typical shade-loving herb, thrives exclusively in quadrats where the average daily solar radiation is less than 1876 Wh/m^2^, and the average daily sunshine duration is below 1.34 h, measured between the spring and autumn equinoxes. In contrast, for 10 other plant species recorded in the study area, species occurrence appeared random, with low or irregular abundance values based on survey and statistical analysis (Figure 2 and Figure S2). Another contributing factor is the relatively high soil moisture and atmospheric humidity in areas with weak solar radiation compared to sunnier regions. This elevated humidity, both above and below ground, creates conditions more favorable for the survival and reproduction of *Nephrolepis auriculata*.

Predicting suitable plant habitats based on solar radiation is highly valuable for applications such as revegetation, rare species protection, and biodiversity maintenance. For instance, ferns can be utilized to prevent soil erosion [66], where their abundance and specific traits play a particularly critical role. For plant species whose abundance is sensitive to solar radiation, the application of digital technology to simulate sunshine distribution in urban environments offers a promising approach. Such simulations can help identify optimal areas for planting and protection, thereby supporting the sustainable management of these species [41,42].

### 2.3. Solar Radiation and Plant Growth Dynamics

**Figure 3:**
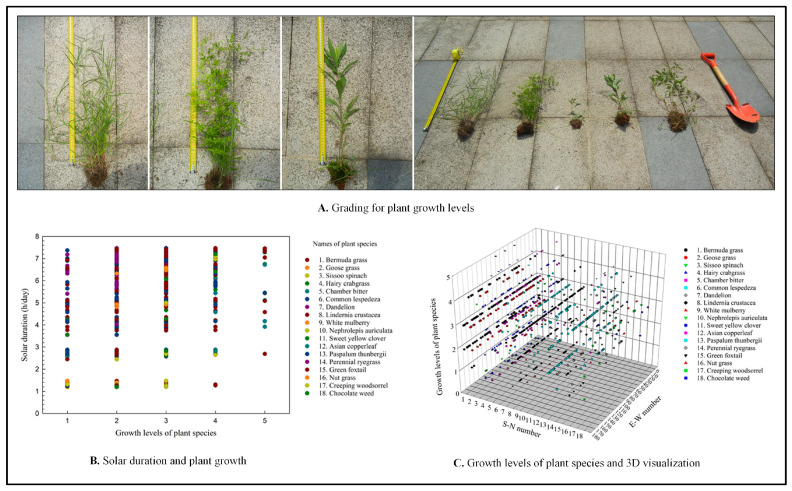
Relationship between solar radiation and plant growth levels.

We used steel tapes and shovels for measurement and sampling. Subsequently, based on the classification criteria established in our experiment, plant growth levels were divided into five grades (1 to 5; Figure 3). During the period from the vernal equinox to the autumnal equinox, the average sunshine duration across the 180 plots ranged from 1.17 to 7.48 h (Table S6 in the Supplementary Material). Sample plants with independent stems were measured using a tape. For creeping species or plants without independent stems, where dry matter measurement posed challenges, on-site judgments were made based on leaf and branch size, with data verification performed through photographs. The survival of plants under limited light conditions is constrained by their light compensation point (LCP). However, additional environmental factors, such as soil moisture and nutrient availability, can influence the LCP, thereby affecting plant growth levels [67,68]. As this is a protected site, the soil composition and moisture content can be considered homogeneous due to the absence of fertilization and irrigation.

In fact, the relationship between solar radiation and soil moisture is quite complex. In comparison to shaded areas, strong sunlight and extended daylight hours tend to increase the evaporation of soil moisture, and plant transpiration also significantly rises. Therefore, in this study site, areas with higher solar radiation may experience soil moisture as a key limiting factor for plant growth, distribution, and diversity. However, it is challenging to separately analyze the effects of these two factors unless soil moisture measurements are taken at regular intervals for each sampling plot.

Our results indicate a positive correlation between plant growth levels and sunshine duration in most grasses of the family *Poaceae*. This correlation may be attributed to the fact that these plants belong to the $C_{4}$ species, which have an enhanced capacity to accumulate carbohydrates with increasing solar radiation [40,69]. In this study, the examined species included *Bermudagrass*, *Hairy crabgrass*, *Paspalum thunbergia*, *Perennial ryegrass*, and *Green foxtail*. Among these, *Bermudagrass*, originally sown as the dominant seed, has been gradually replaced by other species in some quadrats, particularly in areas with low sunshine duration (less than 1.3 h). These findings suggest that grasses of the family *Poaceae* are not well suited for growth in environments with diverse solar radiation levels and are likely to exhibit uneven growth patterns under such conditions.

The same plant species, such as *Goose grass*, *Common lespedeza*, *Dandelion*, *Lindernia crustacea*, and *Mulberry*, show no significant correlation between sunshine duration and growth levels (Figure S3). However, the distribution of *Mulberry* across quadrats appears random, making it challenging to determine whether this pattern is consistent in other areas of the city. In contrast, the analysis revealed a weak negative correlation between the growth level of *Chamber bitter* and sunshine duration in this study area. This plant is a shade-loving species, and its sunlight requirements align with the observed results.

Overall, changes in abiotic environments significantly influence plant species composition and community structure, particularly over large spatial and temporal scales [62,70,71]. Urban areas, with their diverse microenvironments, serve as valuable testing grounds for understanding the factors that drive plant species distribution. However, mitigating human disturbances in such settings is challenging unless diverse types of protected areas are established within the urban built environment.

### 2.4. Additional Drivers of Species Distribution

The urban environment serves as a complex habitat for plant species, shaped by both abiotic and biotic factors, which act as key drivers of plant dispersal. In this study, soil moisture, texture, nutrients, air temperature, and precipitation were consistent across all quadrats, with solar radiation being the only differing abiotic factor. However, the distribution of *Mulberry* and *Dandelion* within the quadrats could not be explained solely by their correlation with solar radiation.

**Figure 4:**
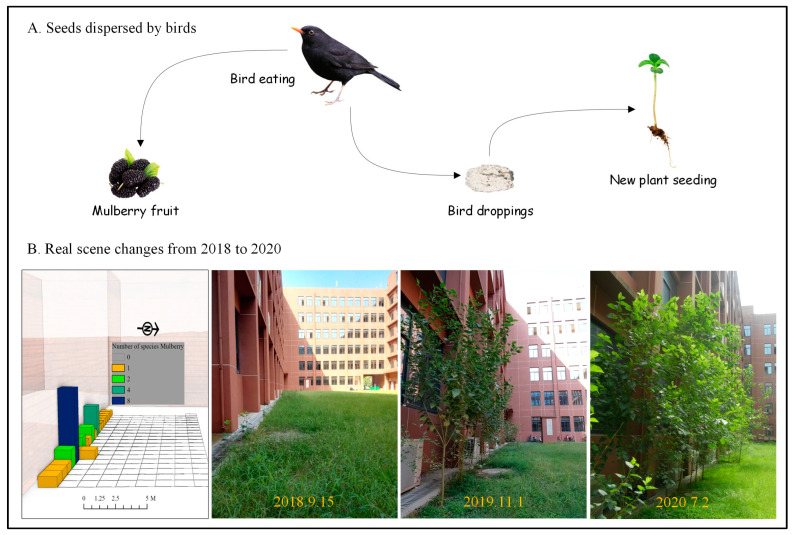
Bird-driven plant species distribution: (**A**) transmission route and (**B**) changes in real scene from 2018 to 2020.

We explored the role of birds in plant dispersal by complementing the data with recordings of bird movements and perching behaviors, which were captured over the past three years using cameras. Building edges and eaves, favored perches for many bird species, were found to accumulate bird droppings containing undigested plant seeds, particularly in the footing areas. Figure 4 illustrates the bird-borne transmission route of *Mulberry*, along with changes in its distribution from 2018 to 2020 under the protection of the experimental site. These findings highlight the crucial role of urban animals, especially birds, in the dispersal of plant species. The interaction between birds and plants promotes mutual benefits, increasing both species abundance and richness in the absence of human interference.

In contrast to plants dispersed by birds, *Dandelion*, another species observed in our study, is primarily dispersed by wind. Affected by swirling winds in the built environment, mature Dandelion seeds tend to fall near the base of buildings. We are currently tracking and observing whether other wind-dispersed plant species exhibit similar distribution patterns.

Our findings show that both *Mulberry* and *Dandelion* were distributed in similar quadrats with respect to solar radiation. However, three years later, as the *Mulberry* tree expanded its canopy, the richness and abundance of *Dandelion* significantly decreased. This change may be due to the shading effect of the plant canopy, which limits solar radiation, or the influence of other ecological factors. Currently, we do not have sufficient evidence to confirm this hypothesis. Moreover, non-natural reserves in urban areas, combined with frequent changes in land use, make it unlikely for climax plant communities to develop in built-up environments.

## 3. Methods and Data

### 3.1. Study Area and Experimental Setup

#### 3.1.1. Site Description

This study was conducted at the urban plant digital simulation and monitoring station, situated on the campus of Jiangxi Normal University. This site is characterized by flat terrain and homogeneous soil, classified as typical red loam soil [72]. The site has been approved by the university and managed and protected by our research team since 2017. This study site does not involve irrigation, fertilization, or other human interventions. Nanchang, where the site is located, lies between 115°27′ and 116°35′ E and 28°09′ and 29°11′ N, in a subtropical monsoon climate zone. The average annual temperature is 17.5 °C, and the average annual precipitation, based on historical data, is approximately 1715 mm. Initially, the area was used as a lawn, with a single perennial herb, Bermudagrass (*Cynodon dactylon* L. Pers.), planted artificially. Following long-term protection by our team and the influence of natural processes, the site’s plant species diversity has significantly increased, with the number of species reaching 18 and exhibiting distinct spatial distribution patterns. In terms of solar radiation, the enclosure and shading effects created by buildings generate a diversified solar radiation environment, thereby establishing a gradient of solar radiation intensity across the site. Detailed plant species names are provided in Table S1 in the Supplementary Materials.

#### 3.1.2. Plot Design and Configuration

The total area of the experimental site is 720 m^2^, predominantly consisting of herbaceous plant species, with standard 2 m × 2 m quadrats established for sampling. The entire survey area is a rectangular plot measuring 18 m by 40 m. Considering the scale of the site and the characteristics of the plant species, a more refined grid system was applied, resulting in a total of 180 survey quadrats. During the survey, a tape measure was used for grid division, and each sample was marked with bamboo stakes and labels. These quadrats serve as permanent monitoring units, forming the basis for plant survey data across multiple years.

To investigate the impact of solar radiation on plant diversity, this study selected 60 sample plots from a total of 180 based on simulated solar radiation values. These plots were then categorized into three groups—Group A, Group B, and Group C—corresponding to the lowest, medium, and highest levels of solar radiation intensity, respectively. Detailed information is provided in Tables S2–S4 in the Supplementary Materials.

### 3.2. Plant Species Survey and Data Collection

#### 3.2.1. Survey Methods and Data Collection Tools

The data for this study were collected using field survey forms, followed by statistical analysis. The survey forms included data fields such as sample identification numbers, photo collection numbers, canopy light intensity values, and recording times. Plant species information covered scientific names, growth conditions, species abundance, coverage, and growth stages.

For woody and herbaceous plants with independent stems, species abundance was determined by counting individual plants. In contrast, for creeping plant species, coverage was assessed based on the grading criteria outlined in Table 1. The biomass was estimated using a simplified method that relates plant body length to biomass. Specifically, a steel tape measure and a digging shovel were used to measure plant length, which was then used to calculate fresh biomass. Plot photographs, recording times, and solar radiation intensity at the plant canopy level were documented using a smartphone equipped with the Light Meter application (Android-based mobile app). This application, originally designed for indoor light measurement, was adapted for use in this study. The structure of the data collection forms is detailed in Table S5 in the Supplementary Materials.

#### 3.2.2. Plant Diversity Indices

This study employs three classical plant diversity indices: the Species Richness Index (R), Simpson’s Diversity Index (D), and the Shannon–Wiener Index (H), to quantitatively assess the distribution and diversity of plant species.

(1) Species Richness Index (R)

The Species Richness Index (R) is the most straightforward method for measuring plant diversity, calculated as the total number of plant species within a given plot. This index reflects only the species count and does not account for species dominance or abundance. The formula for calculating the Species Richness Index is as follows:

R=(n1+n2+…)N

where $n_{1}$ and $n_{2}$ represent the number of individuals of the first and second plant species in the plot, respectively, and N is total number of plant species within the study area.

(2) Simpson’s Index of Diversity (D)

Simpson’s Diversity Index (D) is a formula that is widely used to evaluate species diversity within an ecosystem. The value of this index ranges from 0 to 1, with lower values indicating higher species diversity. In this study, we adopt a modified version of this formula to enhance its interpretability and intuitive understanding. However, it is important to note that the mathematical meaning of this modification is opposite to that of the original formula. The specific formula is as follows:

$$D=1-\sum Ni(Ni-1)/\left[N(N-1)\right]$$

where Ni represents the number of individuals of the ith species, and N is the total number of individuals within the study area.

(3) Shannon–Wiener Index (H)

The Shannon–Wiener Index (H), derived from information theory, is one of the most commonly used indices in the ecological studies of plant diversity. It measures the degree of disorder or uncertainty in the distribution of species, with higher values indicating greater uncertainty. The formula for the Shannon–Wiener Index is as follows:

$$H=-\sum \left[\left(\frac{n_{i}}{N}\right)\times In\left(\frac{n_{i}}{N}\right)\right]$$

where $n_{i}$ is the number of individuals of the ith species, N is the total number of individuals within the study area, and In denotes the natural logarithm.

By utilizing these indices, this study can assess plant species diversity and distribution from multiple perspectives.

#### 3.2.3. Data Processing and Integration

Data from the test site were collected and digitized using Microsoft Excel. The vector grid for each quadrat was generated using ArcGIS Pro software, with coordinate registration and the creation of a database. The Excel data were subsequently imported into the GIS vector spatial data using the ‘Add Join’ function in the GIS attribute table. Similarly, solar radiation data were integrated into the spatial attribute table of the quadrats using the same method. For the entire study, data calculations, analysis, visualization, and graphing were conducted using MATLAB 2018 (MathWorks), SigmaPlot 14 (SYSTAT), and ArcGIS Pro 2.5 (ESRI).

### 3.3. Solar Radiation Simulation and Calibration

#### 3.3.1. Solar Radiation Simulation

Solar radiation is a fundamental energy source for primary productivity on Earth. However, most available solar radiation data are derived from meteorological observation stations, making it challenging to obtain high-resolution data across diverse spatiotemporal dimensions, particularly at the meter scale in urban built environments. Advances in digital tools, including specialized software, computational models, and high-performance PCs, have enabled the quantitative modeling of solar radiation [42,73,74,75]. In this study, the Solar Analyst module in ArcGIS Pro was employed to simulate solar radiation, building on its proven stability in our previous research [40,41,42]. Given the relatively small simulation area and the need to balance computational time and efficiency, a finer grid resolution of 0.1 m × 0.1 m was implemented during the conversion of vector data to raster data. Detailed parameter settings for the Solar Analyst module are presented in Table 2.

Moreover, as most plant species at the survey site are annual or perennial deciduous plants, the time interval for the solar simulation was selected between the vernal and autumnal equinoxes, which is the most critical and effective period for plant growth and development. The solar radiation simulation outputs include total solar radiation (WH/m^2^), direct solar radiation (WH/m^2^), and solar duration (hours). The center of each quadrat was designated as a target point, and the simulated solar radiation results were extracted into the corresponding quadrat dataset using the ‘Extract Values to Points’ function in the Geoprocessing Tools.

#### 3.3.2. Model Calibration and Validation

**Figure 5:**
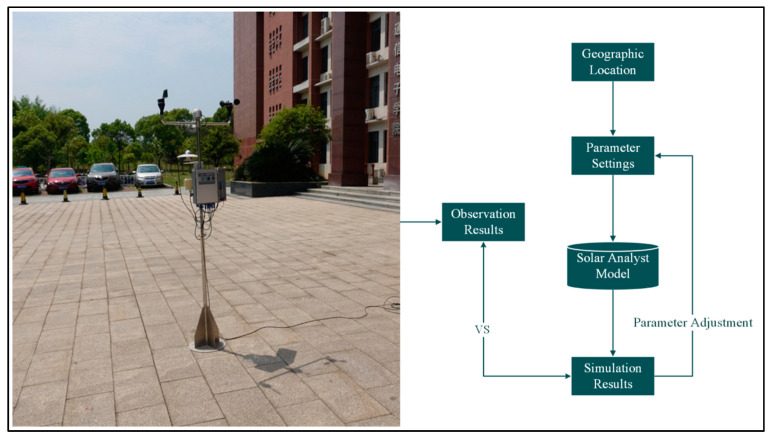
Calibration of the model using the radiometer from the Microclimate Environment Test System.

To ensure the accuracy of the simulation results in Nanchang, model calibration was performed using instrumental measurements from open test sites. The radiometer module of the Microclimate Environment Test Systems (No. JRT13) was used to measure solar radiation, which was then used to adjust the model’s output (Figure 5). The calibration formula is as follows [40]:

(1) $$R_{solar}=\frac{R_{test}}{R_{simulation}}\times R_{model}$$

where $R_{solar}$ represents the solar radiation at the study site, $R_{test}$ denotes the solar radiation measured at the test point using the instrument, $R_{simulation}$ is the simulated solar radiation value generated by the Solar Analyst module in ArcGIS Pro 2.5, and $R_{model}$ refers to the maximum value from the model simulation.

At the same location, the meteorological station measurement was conducted around 11:00 a.m. on a day in July (Figure 5). To align with this, the solar radiation model was set to the same time, with a 1 h time interval selected. Finally, the values derived from model (1) were used as coefficients, and in the GIS data table, the formula calculator module was applied to adjust the solar radiation simulation values extracted from the 180 plots for overall numerical calibration.

### 3.4. Experimental Design and Research Process

**Figure 6:**
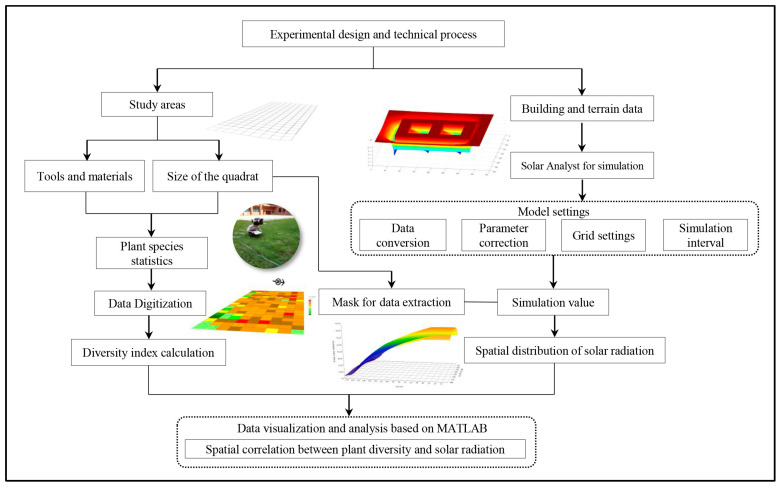
Experimental design and technical processing framework.

Based on the characteristics of the experimental site, as well as the materials, tools, and techniques utilized, the experimental design and research process are organized into several key steps. These include the establishment of study quadrats and the selection of appropriate tools for data collection, followed by the preparation of building and terrain data, the generation of vector models, and the simulation of solar radiation. The next step involves processing plant species and solar radiation data, which includes digitization and integration through GIS to assess the spatial distribution of plant species and their correlation with solar radiation. Finally, data calculation and visualization analysis are performed. A detailed overview of the research process is presented in Figure 6.

## 4. Conclusions and Future Prospects

Urban built-up areas should be recognized as key areas for biodiversity conservation in the context of accelerated urbanization. However, cities are complex systems, and studies on how solar radiation influences the spatial distribution of plant species remain limited. While the existing literature has highlighted the impact of built-up area density and function on plant species patterns, with conclusions suggesting that densely built-up areas are the most powerful predictors of species composition [10], there is still a need for more quantitative data on solar radiation to further investigate and validate this topic.

In this study, we report for the first time, based on three years of monitoring and survey data at the test site, that solar radiation drives the distribution of urban plant species. To assess the spatial distribution of plant diversity, we employed three species diversity indices: the Species Richness Index (R), Simpson’s Diversity Index (D), and the Shannon–Wiener Index (H). Solar radiation data for the research site were obtained through digital simulation technology, supplemented by on-site model calibration.

Our findings lead to the following conclusions:

(1) In urban built-up areas, the distribution patterns of plant species, including species composition and diversity, is associated with solar radiation.
(2) Based on the Species Richness Index (R), the highest average richness is observed in quadrats with lower solar radiation. Similarly, the highest average diversity, as measured by the Simpson’s Diversity Index (D), is found in quadrats with higher solar radiation. While the Diversity Index (D) and the Shannon–Wiener Index (H) show slight differences, D is more sensitive than H for evaluating species distribution.
(3) Solar radiation impacts the abundance and growth levels of plant species, but the correlation varies significantly across species. Additionally, our data suggest that, beyond solar radiation, birds and wind play important roles in the spatial distribution of certain species.

In the future, we will continue to track the spatiotemporal changes in plant species distribution driven by solar radiation, utilizing more refined data.

Under the combined influence of long-term solar radiation, soil moisture, and other environmental factors, the dynamics of the nutrient distribution of different plant species will gradually stabilize. By tracking the migration patterns of these species and their eventual establishment of stable communities, a more comprehensive assessment of their ecological amplitude in adapting to solar radiation can be achieved.

However, compared to solar radiation, the definitive role of soil moisture requires further in-depth investigation. This is particularly true for hydrophilic and shade-tolerant plant species, whose ecological distribution ranges may be significantly influenced by variations in soil moisture. According to precipitation data from Nanchang, the annual rainfall is approximately 1715 mm. This level of precipitation suggests that, for most plant species, soil moisture is unlikely to be a critical limiting factor for their natural distribution.

The experimental site will serve as a platform for continuous monitoring, focusing on changes in species richness, diversity, growth trends, and other key characteristics of the plots over time. As the study advances, we aim to capture the long-term dynamics of plant communities and their responses to variations in solar radiation, thereby providing deeper insights into the mechanisms driving plant distribution in urban environments.

In an optimal scenario, if the ecological niches (i.e., the range of environmental factors such as solar radiation and soil moisture) required by each plant species could be accurately measured, then, by combining these fundamental data with GIS technology, it would be possible to predict the spatial distribution of species in built environments with a high degree of accuracy.

## Figures and Tables

**Table 1:** Grading criteria for plant coverage based on visual estimation.

Level	Judgmental Features	Coverage Percentage (%)
I	Plants cover the entire quadrat; plant bodies are consistently connected.	76–100
II	Plant coverage is high, but individuals are not fully connected.	51–75
III	diversity plant coverage, not yet half of the quadrat.	26–50
IV	Low plant coverage, with scattered plant appearances.	6–25
V	Plants are rare, with very low coverage.	<5

**Table 2:** Parameter settings for the Solar Analyst model.

Input Raster	Cell Size	Latitude	Time Configuration	Transmittivity	Diffuse Proportion
Building footprint	0.1 m × 0.1 m	N 28.7	From spring to autumn	0.5	0.3

## Data Availability

Data available on request due to restrictions.

## Supplementary Materials

The following supporting information can be downloaded at: https://www.mdpi.com/article/10.3390/plants14040539/s1.
