# Supplementary material for: Solar Radiation Drives the Plant Species Distribution in Urban Built-Up Areas
Source: Plants (Basel). 2025 Feb 10;14(4):539. doi: 10.3390/plants14040539 (PMC11859860; doi:10.3390/plants14040539)
Supplement: Supplementary file 1 [file plants-14-00539-s001.zip › plants-3394597-supplementary.pdf]

Solar Radiation Drives the Plant Species Distribution in Urban Built-Up Areas

Heyi Wei, Bo Huang, Mingshu Wang, Xuejun Liu

Correspondence: weihy@whu.edu.cn

Table S1. Corresponding Names of Plant Species

SPECIES NO.	ENGLISH NAME	SCIENTIFIC NAME	GROWTH HABIT
1	Bermuda grass	Cynodon dactylon	Perennial herb
2	Goose grass	Eleusine indica (L.) Gaertn.	Annual herb
3	Sissoo spinach	Alternanthera sessilis (L.) DC.	Perennial herb
4	Hairy crabgrass	Digitaria sanguinalis (L.) Scop.	Annual herb
5	Chamber bitter	Phyllanthus urinaria L.	Annual herb
6	Common lespedeza	Kummerowia striata	Annual herb
7	Dandelion	Taraxacum mongolicum Hand.-Mazz.	Perennial herb
8	Lindernia crustacea	Lindernia crustacea (L.)F. Muell	Annual herb
9	White mulberry	Morus alba L.	Deciduous tree
10	Nephrolepis auriculata	Nephrolepis auriculata (L.) Trimen	Perennial herb
11	Sweet yellow clover	Melilotus officinalis (L.) Pall.	Biennial herb
12	Asian copperleaf	Acalypha australis L.	Annual herb
13	Paspalum thunbergii	Paspalum thunbergii Kunth ex steud.	Perennial herb
14	Perennial ryegrass	Lolium perenne L.	Perennial herb
15	Green foxtail	Setaria viridis (L.) Beauv.	Annual herb
16	Nut grass	Cyperus rotundus L.	Perennial herb
17	Creeping woodsorrel	Oxalis corniculata L.	Perennial herb
18	Chocolate weed	Melochia corchorifolia Linn.	Annual herb

Table S2. The Quadrat with the Lowest Solar Radiation

Quadrat coding	Numbers of species	R richness	D diversity	H diversity
1	8	0.444444444	0.816993464	0.360413429
18	8	0.444444444	0.816993464	0.360413429
19	7	0.388888889	0.862745098	0.367290626
36	12	0.666666667	0.568627451	0.270310072
37	11	0.611111111	0.640522876	0.300957852
54	7	0.388888889	0.862745098	0.367290626
55	10	0.555555556	0.705882353	0.326548147

72	8	0.444444444	0.816993464	0.360413429
73	6	0.333333333	0.901960784	0.366204096
90	7	0.388888889	0.862745098	0.367290626
91	5	0.277777778	0.934640523	0.355814957
108	6	0.333333333	0.901960784	0.366204096
109	6	0.333333333	0.901960784	0.366204096
126	7	0.388888889	0.862745098	0.367290626
127	9	0.5	0.764705882	0.34657359
144	6	0.333333333	0.901960784	0.366204096
145	8	0.444444444	0.816993464	0.360413429
162	6	0.333333333	0.901960784	0.366204096
163	10	0.555555556	0.705882353	0.326548147
180	4	0.222222222	0.960784314	0.334239422

Table S3. The Quadrat with Medium Solar Radiation

Quadrat coding	Numbers of species	R richness	D diversity	H diversity
5	5	0.277777778	0.934640523	0.355814957
14	5	0.277777778	0.934640523	0.355814957
23	4	0.222222222	0.960784314	0.334239422
32	4	0.222222222	0.960784314	0.334239422
41	5	0.277777778	0.934640523	0.355814957
50	6	0.333333333	0.901960784	0.366204096
59	6	0.333333333	0.901960784	0.366204096
68	5	0.277777778	0.934640523	0.355814957
77	5	0.277777778	0.934640523	0.355814957
86	6	0.333333333	0.901960784	0.366204096
95	5	0.277777778	0.934640523	0.355814957
104	6	0.333333333	0.901960784	0.366204096
113	4	0.222222222	0.960784314	0.334239422
122	4	0.222222222	0.960784314	0.334239422
131	6	0.333333333	0.901960784	0.366204096
140	5	0.277777778	0.934640523	0.355814957
149	5	0.277777778	0.934640523	0.355814957
158	6	0.333333333	0.901960784	0.366204096
167	6	0.333333333	0.901960784	0.366204096
176	5	0.277777778	0.934640523	0.355814957

Table S4. The Quadrat with the Highest Solar Radiation

Quadrat coding	Numbers of species	R richness	D diversity	H diversity
9	8	0.444444444	0.816993464	0.360413429
10	4	0.222222222	0.960784314	0.334239422
27	5	0.277777778	0.934640523	0.355814957
28	5	0.277777778	0.934640523	0.355814957
45	5	0.277777778	0.934640523	0.355814957
46	6	0.333333333	0.901960784	0.366204096
63	6	0.333333333	0.901960784	0.366204096
64	5	0.277777778	0.934640523	0.355814957
81	5	0.277777778	0.934640523	0.355814957
82	5	0.277777778	0.934640523	0.355814957
99	6	0.333333333	0.901960784	0.366204096
100	6	0.333333333	0.901960784	0.366204096
117	7	0.388888889	0.862745098	0.367290626
118	5	0.277777778	0.934640523	0.355814957
135	5	0.277777778	0.934640523	0.355814957
136	6	0.333333333	0.901960784	0.366204096
153	5	0.277777778	0.934640523	0.355814957
154	6	0.333333333	0.901960784	0.366204096
171	5	0.277777778	0.934640523	0.355814957
172	5	0.277777778	0.934640523	0.355814957

Table S5. Questionnaire Design for Plant Species Survey

No. of plot:		Full light (LUX):		No. of photo:	
Investigator name:		Canopy light of plot (LUX):		Data and Time:	
Plant names		Coverage (abundance)<br>(1 / 0.8 / 0.6 / 0.4 / 0.2)		Growth levels<br>(I、II、III、IV、V)	
Species 1					
Species 2					
Species 3					
Species 4					
Species 5					
Species 6					
Species 7					
Species 8					
Species 9					
Species 10					

Figure S1. 3D Visualization of Plant Species Abundance Across Quadrats Under Varying Solar Radiation Levels

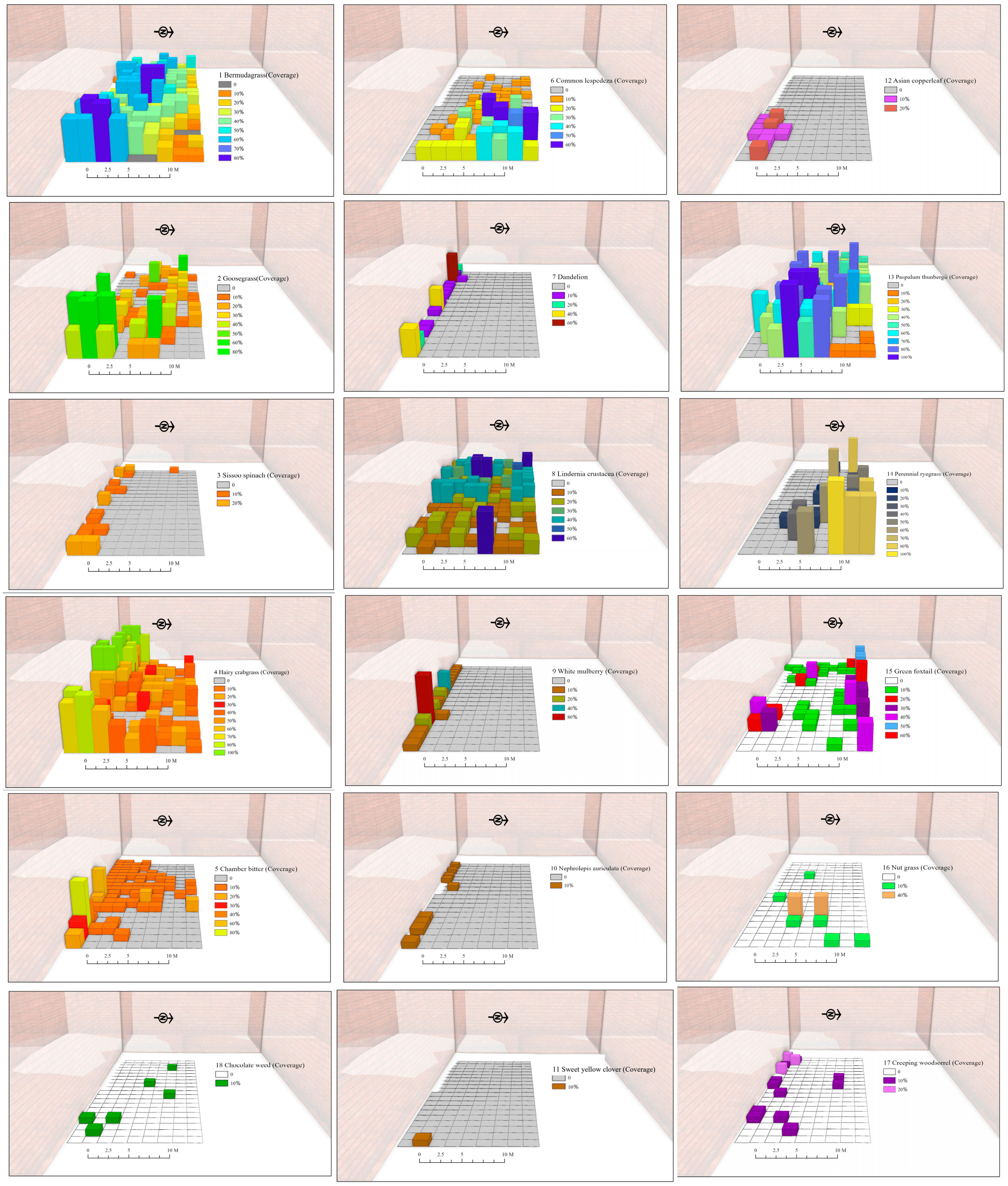

Figure S2. Abundance Simulation of 18 Plant Species Based on Digital Models

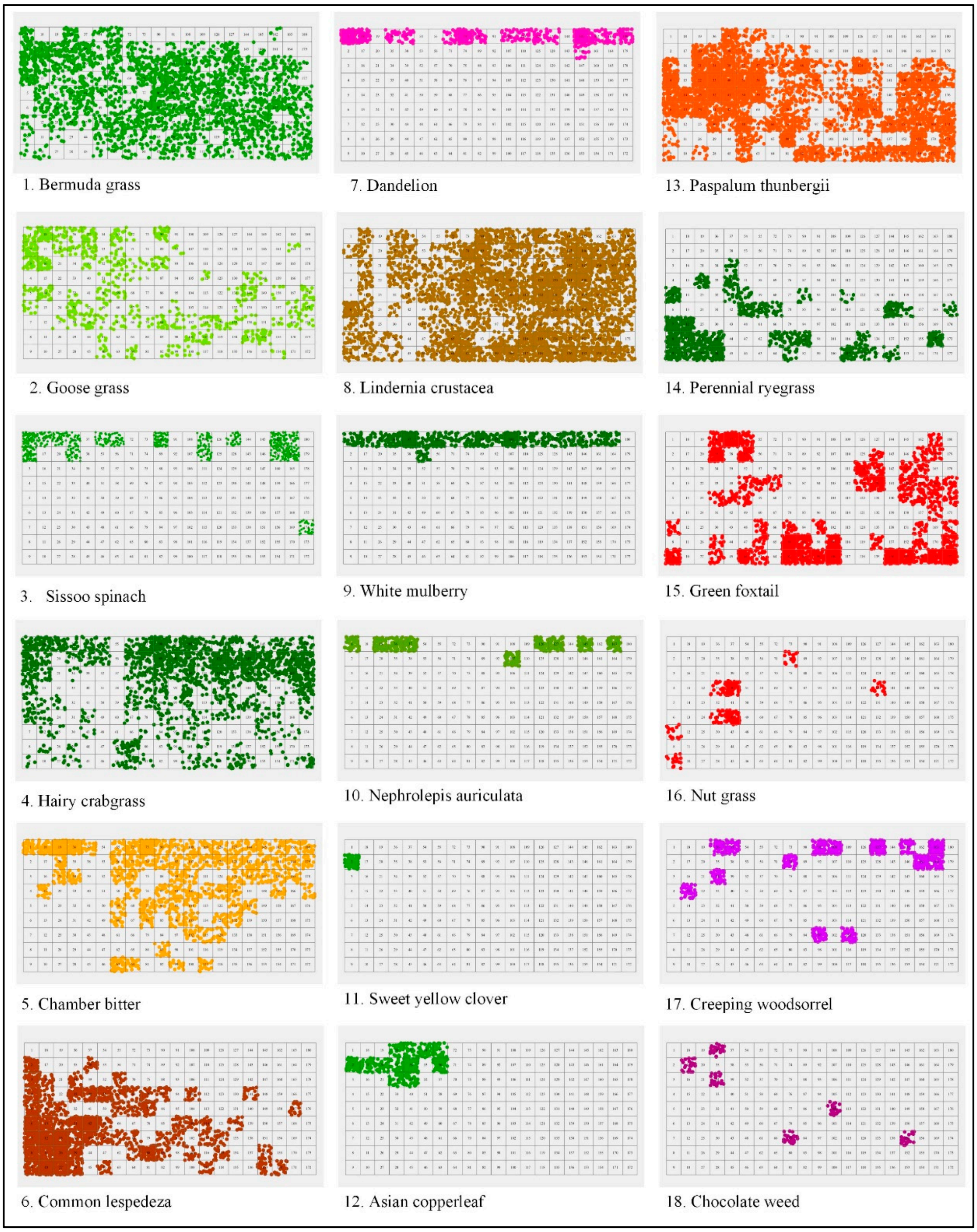

Figure S3. Correlation Analysis of Abundance (Coverage), Growth, and Sunshine Duration (a part of the plant species)

Bermuda grass (No. 1)

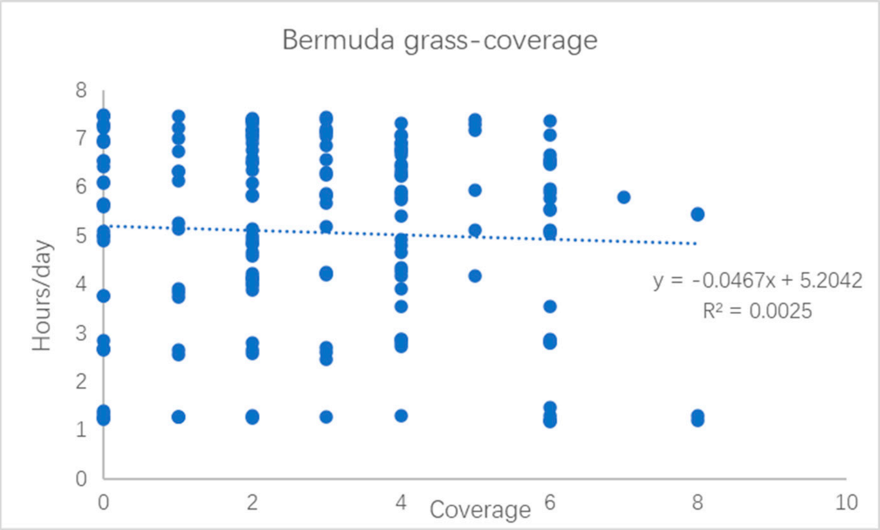

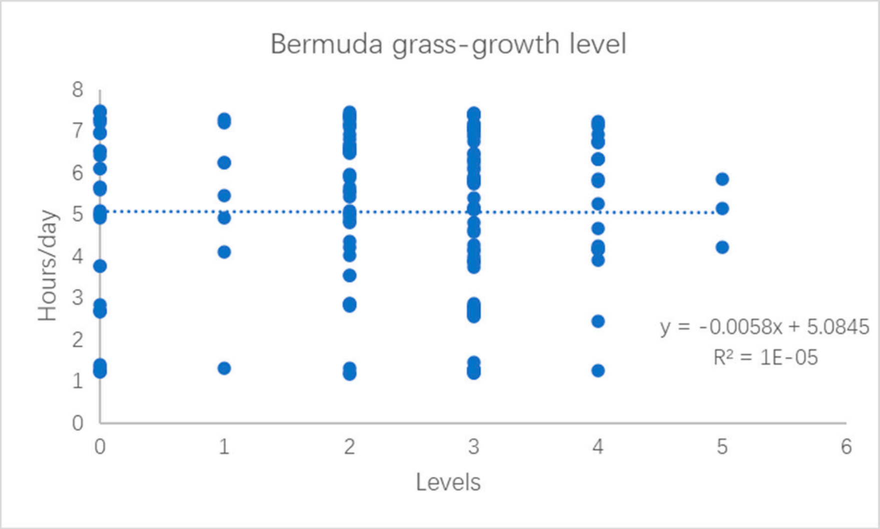

Goose grass (No. 2)

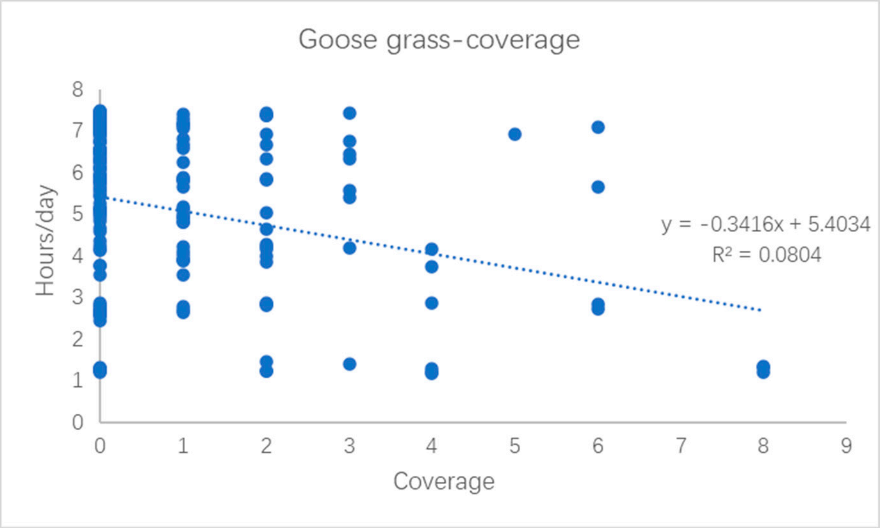

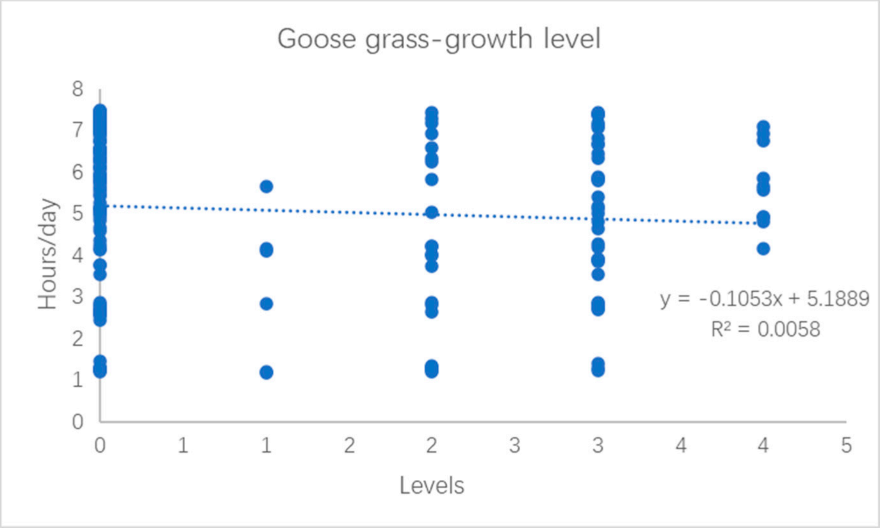

Hairy crabgrass (No. 4)

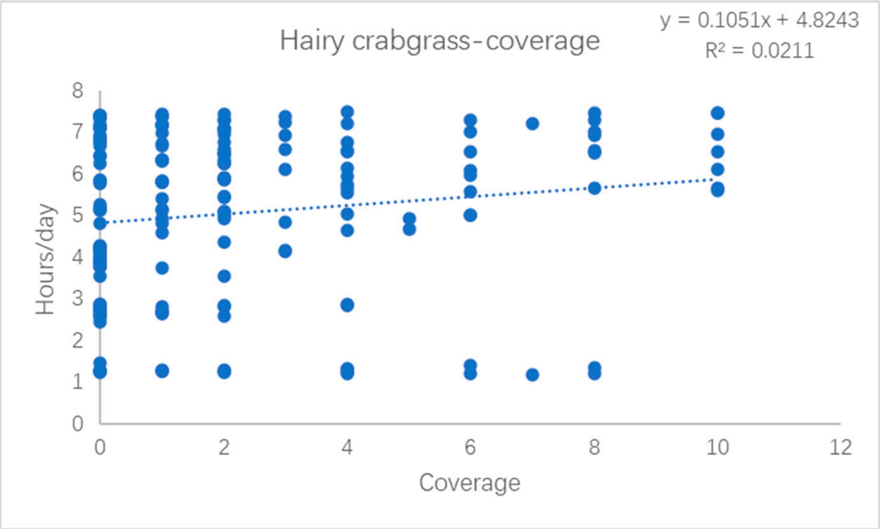

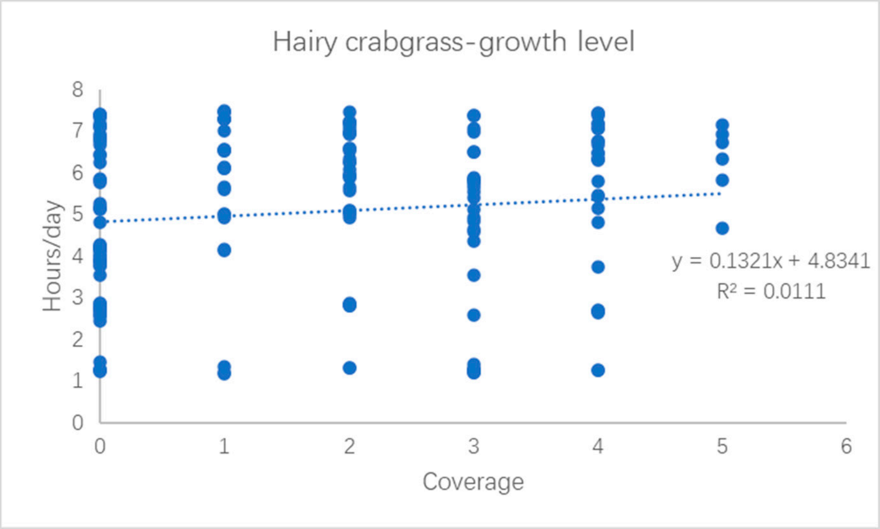

Dandelion (No. 7)

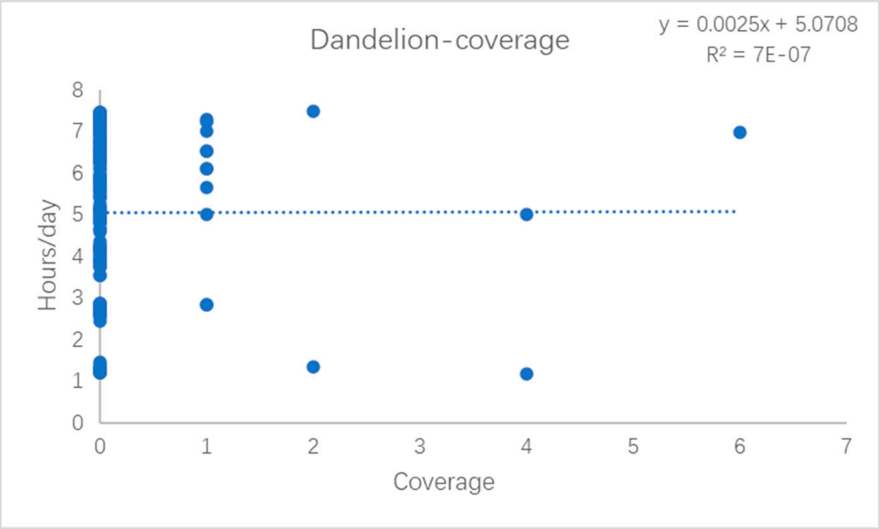

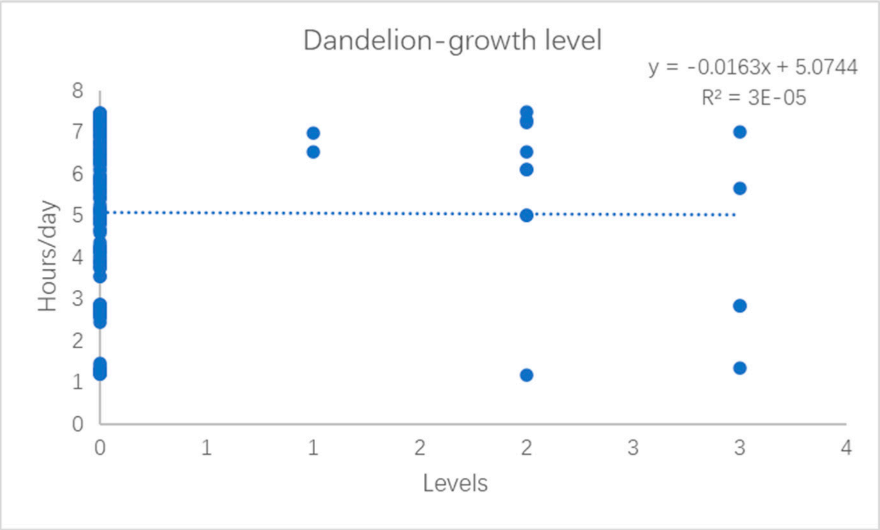

Lindernia crustacea (No. 8)

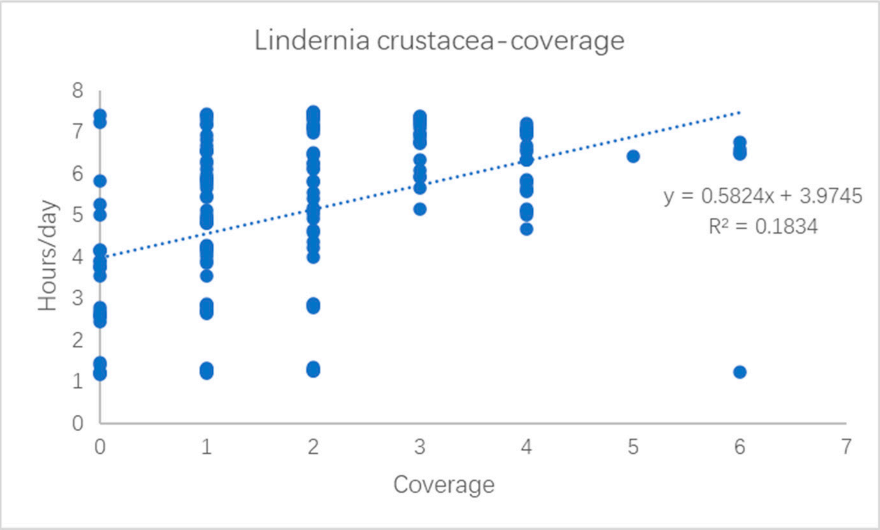

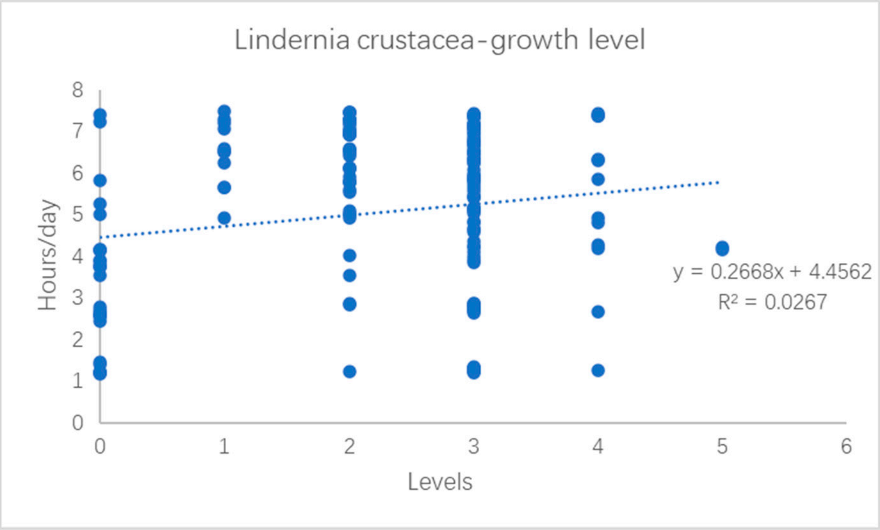

Paspalum thunbergia (No. 13)

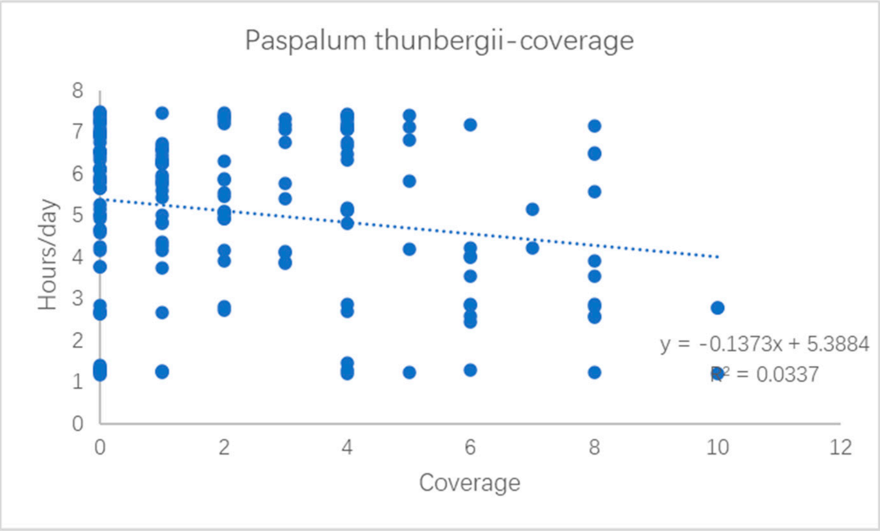

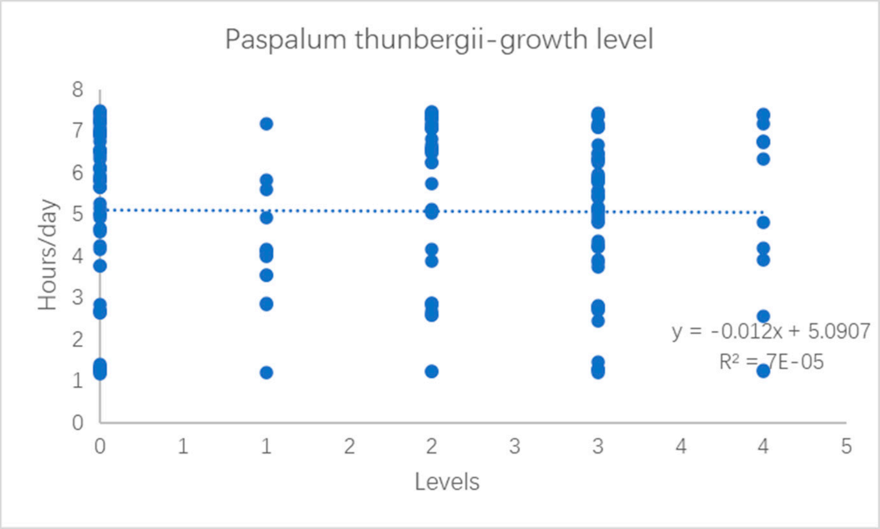

Perennial ryegrass (No. 14)

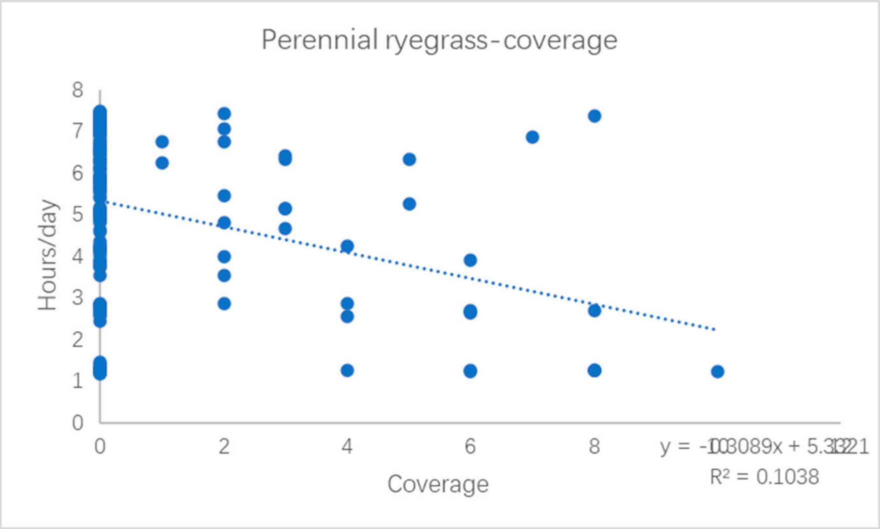

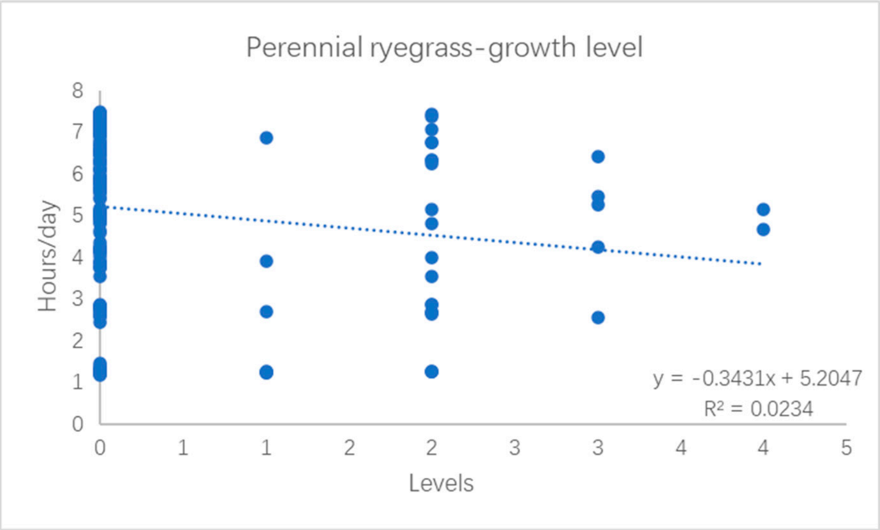

Green foxtail (No. 15)

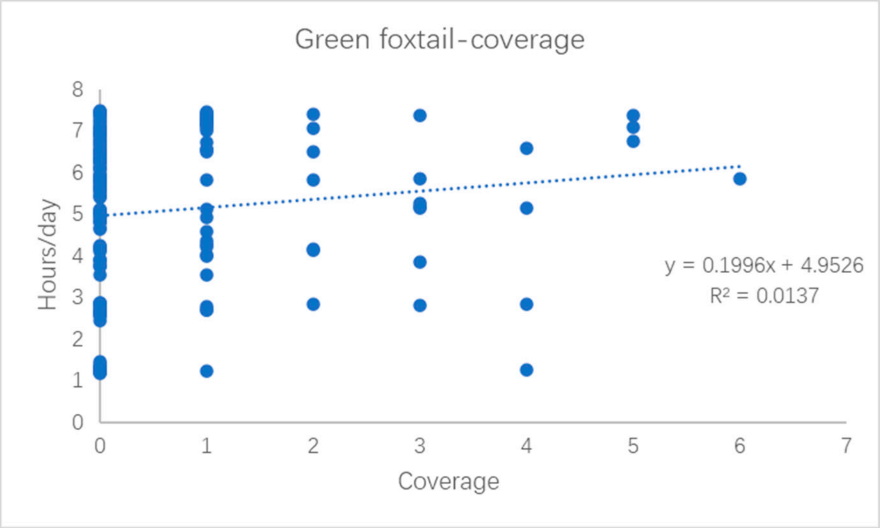

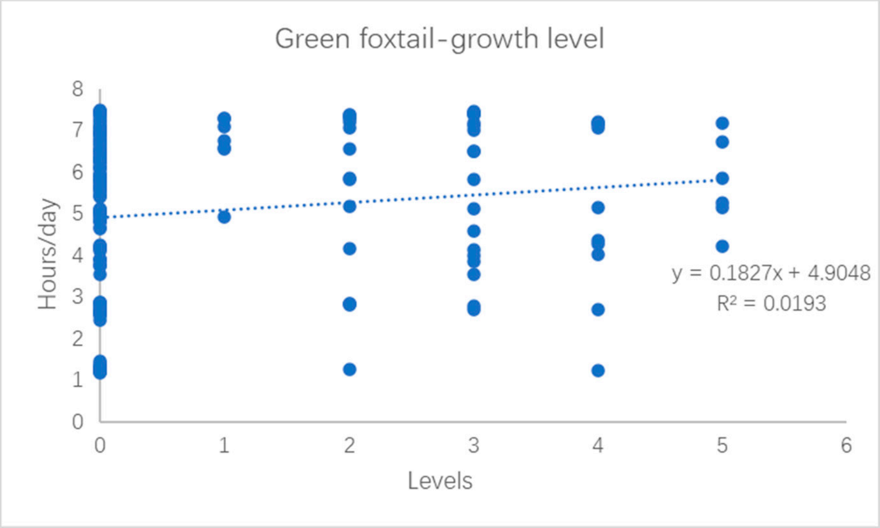

Creeping woodsorrel (No. 17)

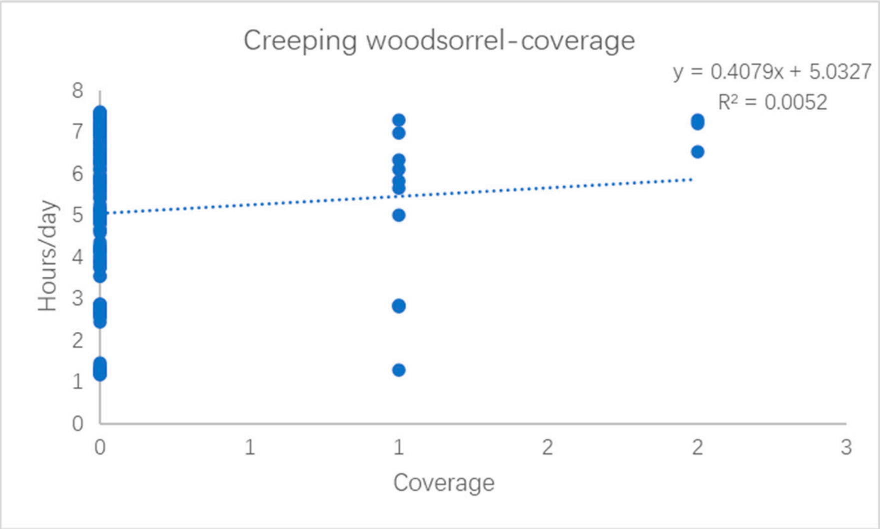

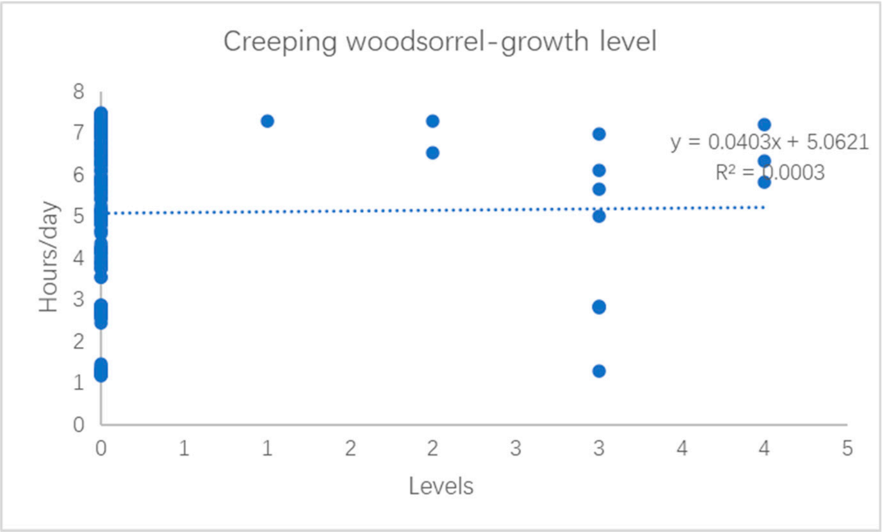

Figure S4. Relationship Between Solar Radiation and Plant Growth Levels; B.Solar duration and plant growth

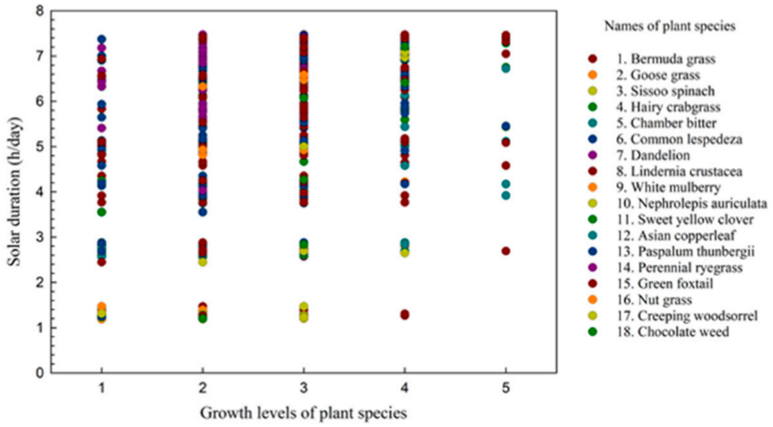

Figure S5. Relationship Between Solar Radiation and Plant Growth Levels; C. Growth levels of plant species and 3D visualization

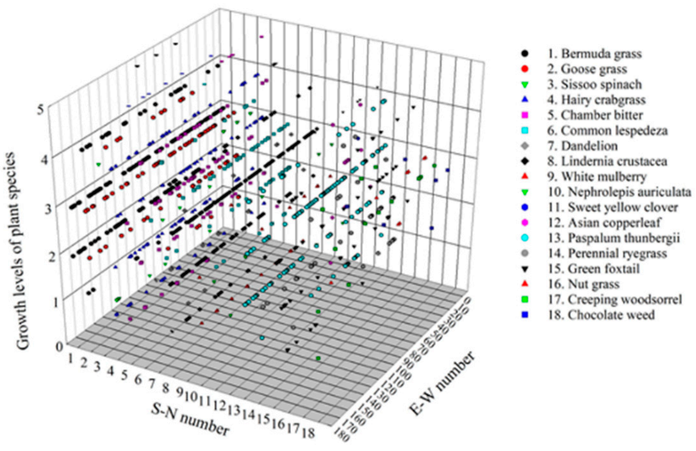

Table S6. Quadrat Coding and Solar Radiation from the Spring Equinox to the Autumn Equinox

Quadrat coding	Total solar radiation (WH⁄m^2)	Direct solar radiation(WH⁄m^2)	Total sunshine duration(hours)	Average hours(H/day)
1	147616.2344	77274.875	230.3556671	1.265690479
2	296119.1563	220723.625	471.8262329	2.592451829
3	424217.7813	343561.5625	685.5435791	3.766722962
4	513626.4063	427996.5313	846.0186157	4.648453933
5	588757.6875	498467.7813	989.5883789	5.437298785
6	637399	544352.25	1080.233643	5.935349684
7	686629.5625	590844.5625	1177.722168	6.471000923
8	710098.5625	612394.125	1227.036987	6.741961469
9	711139.125	612919.3125	1230.196777	6.759322952
10	735148.8125	633543.5	1262.178101	6.935044509
11	733120.5625	632428.0625	1258.35144	6.914018903
12	711888.8125	613072.6875	1213.969604	6.670162662
13	672973.75	576054	1135.259155	6.237687666
14	607047.6875	513956.4375	1011.097595	5.555481292
15	536260	447609.3125	876.8127441	4.81765244
16	437554.3438	354481.2813	698.7099609	3.839065719
17	328628.875	250380.5781	516.8527222	2.839850122
18	151085.2031	78628.35156	225.5866852	1.239487281
19	152572.6719	78574.00781	218.7045288	1.201673235
20	330427.7188	250431.375	510.3329773	2.804027348
21	449977.3438	364683.75	712.6469727	3.915642707

22	550797.375	459763.8125	895.8390503	4.922192584
23	621680.125	526368.1875	1027.463867	5.645405864
24	680470.875	581855.0625	1138.458862	6.255268474
25	734547.625	633000.0625	1246.355835	6.848108983
26	751975.125	648750.625	1282.950928	7.049180922
27	754006.0625	649637.1875	1286.987427	7.071359488
28	768857.6875	662536.375	1309.105347	7.19288652
29	767230.875	661758.4375	1305.787964	7.174659142
30	749389.8125	645648.5	1267.719971	6.965494345
31	692821	592294.75	1153.280518	6.336706141
32	634835.5	537479.5625	1044.06189	5.736603789
33	555699.5625	463166.8125	897.2377319	4.929877648
34	465377.4063	377971.0625	733.7393188	4.031534719
35	317279.5313	236360.6094	482.502655	2.651113489
36	158168.6094	82678.25781	220.4685822	1.211365836
37	163271.2969	86381.42188	223.5222626	1.2281443
38	321740.9688	239626.75	484.2793579	2.660875593
39	474429.2813	385619.3125	747.4150391	4.106676039
40	564208.4375	470264.7188	908.6713257	4.992699592
41	638742.3125	540256.25	1047.666992	5.756412045
42	704038.25	601733.6875	1169.697021	6.426906711
43	755093.4375	649837.875	1271.982788	6.988916418
44	779371.5	672164.0625	1324.633057	7.278203608
45	780315.3125	672549.0625	1327.108154	7.291803046
46	789351.125	680466.4375	1339.666626	7.360805637
47	788337	680033.25	1338.75708	7.355808132
48	765304.4375	658955.9375	1289.019531	7.082524897
49	713130.9375	609436.5625	1182.733032	6.498533144
50	651369.625	551474.6875	1065.254028	5.853044112
51	577190.9375	481804.0625	931.0534668	5.115678389
52	482284.125	392374.1563	759.3145752	4.172058105
53	326808.2813	243825.2031	489.6433716	2.690348196
54	172672.7969	94704.875	236.0163574	1.296793173
55	171891.2188	93505.74219	229.9810638	1.263632219
56	328936.6875	245397.5	488.6576233	2.684931996
57	486357.375	395679.7813	762.8884277	4.191694658
58	579960	484029.6563	930.4894409	5.112579346
59	653877.5	553396.1875	1063.345459	5.842557467
60	717714.0625	613437.5	1187.843262	6.526611328
61	770485.875	663388.9375	1295.397949	7.11757115
62	792627.3125	683685.875	1341.429565	7.370492118
63	794079.125	684330.625	1343.407471	7.381359729
64	799634.25	689426.375	1356.998047	7.456033225

65	798585	689023.875	1355.510986	7.447862562
66	777119	669380.1875	1312.115112	7.209423694
67	722464.375	617762.1875	1198.460938	6.584950206
68	658634.25	557753.125	1074.846313	5.905748975
69	584986.875	488474.25	942.104248	5.176396967
70	489354.2813	398237.0313	769.076416	4.225694593
71	350369.6563	265737.6875	523.928772	2.878729516
72	176198.1875	97361.67969	238.628479	1.311145489
73	175611.7813	96800.78125	237.1571655	1.303061349
74	332649.2188	248586.6875	497.1668701	2.7316861
75	489313.125	398046	768.3895874	4.22192081
76	574472.9375	478481.875	925.519104	5.085269802
77	647999	547769.0625	1055.932373	5.801826226
78	720024.0625	615430.4375	1192.917725	6.554492992
79	778391.4375	670508.5	1315.445679	7.227723509
80	799497.1875	689816.8125	1357.691284	7.459842221
81	800621.1875	690334.75	1359.641724	7.470558921
82	797157.9375	687043.5625	1351.033081	7.423258687
83	795988.4375	686657.0625	1350.023438	7.417711195
84	774621.25	667024.25	1306.62561	7.179261595
85	720772.125	616167.125	1195.568726	6.569058932
86	656144.6875	555450.125	1069.09668	5.874157581
87	582624.6875	486257.5	936.6514282	5.146436419
88	491390	400286.4063	773.0556641	4.247558594
89	349021.8125	264495.8125	521.1791992	2.863621974
90	176130.4531	97023.67188	238.1553345	1.308545794
91	172006.7969	93651.5	233.3299561	1.282032726
92	327848.75	244425.0625	488.4774475	2.683942019
93	479051.125	388832.625	752.4396362	4.134283716
94	579520.125	483617.6563	934.2477417	5.13322935
95	646901.8125	546895.6875	1054.629639	5.794668344
96	716940.375	612710.625	1189.244629	6.534311148
97	769570.0625	662565.25	1297.972168	7.131715209
98	792047.875	683171.5	1345.712769	7.394026201
99	793176.6875	683383.8125	1345.398926	7.39230179
100	786910.625	678165.625	1339.082153	7.357594249
101	783520.6875	675594.1875	1332.293091	7.320291708
102	762793.5	656696	1287.914551	7.076453576
103	709927	606644.8125	1179.212524	6.479189695
104	646698.1875	547155.25	1057.643921	5.811230335
105	574399.5625	479359.9375	928.6242676	5.102331141
106	481639.0938	391969.9375	759.0305176	4.170497349
107	342524.8438	259172.1563	515.7487793	2.833784502

108	167973.7969	90234.84375	230.4711304	1.266324892
109	162670.6406	85914.60156	225.9362183	1.241407793
110	342876.875	260260.0156	521.9569702	2.867895441
111	477229.875	388560.25	755.6488647	4.151916839
112	566493.375	472667.0625	917.008606	5.038508824
113	645338.5625	546488.4375	1061.804443	5.834090348
114	700643.875	598729.5625	1167.121826	6.412757287
115	753270.125	648395.625	1274.421021	7.002313299
116	776017	669222.125	1323.849365	7.273897611
117	776718.1875	669486.125	1325.38147	7.282315768
118	764173.5625	658449.4375	1306.305664	7.177503649
119	762955.6875	657923.3125	1303.588257	7.16257284
120	741195.8125	638269.6875	1258.297363	6.913721776
121	688649.4375	588643.8125	1151.172485	6.325123546
122	624291.125	527878	1029.875488	5.658656529
123	559740.75	467314	910.6781616	5.003726163
124	457467.8125	370769.5625	724.1616821	3.978910341
125	333624.8438	252366.2969	510.8800964	2.807033497
126	156249.9688	80788.96875	220.4355469	1.211184323
127	147613.0938	74053.14844	216.0168152	1.186905578
128	326783.3125	247280.7344	508.3048706	2.792883904
129	447076.7813	362216.9375	713.2301636	3.918847053
130	546671.25	456054.5625	893.4984741	4.909332275
131	617532.625	522635.2813	1027.006592	5.642893362
132	676082.4375	577880.6875	1137.593262	6.250512427
133	725965.0625	625158.9375	1238.876221	6.807012202
134	747219.5	644534.9375	1284.088013	7.055428641
135	749334.875	645486.3125	1288.573486	7.080074101
136	730301.4375	629115.9375	1262.013306	6.934139042
137	727300.1875	627191.625	1256.647827	6.904658391
138	706346.75	608017.1875	1211.57373	6.656998519
139	659066.125	563103	1115.55896	6.129444835
140	601368.8125	508844.75	1007.283569	5.534525106
141	532014.625	443812.9688	875.0531006	4.807984069
142	440003.5625	357044.1875	708.3278198	3.891911098
143	321609.5938	243735.0938	507.9946289	2.79117928
144	152139.6875	79277.85156	231.6207123	1.272641276
145	144966.3281	74970.65625	231.7548828	1.273378477
146	290994.5	216057.9375	468.2091675	2.572577843
147	419614.125	339427.5313	682.4187012	3.749553303
148	512284.8438	426987.8125	849.4934692	4.667546534
149	582129.1875	492506.2188	984.359375	5.408567995
150	635074.75	542429.3125	1084.011108	5.956104991

151	683183.3125	587791.25	1180.585083	6.486731225
152	703102.125	606100.5	1223.741089	6.723852137
153	705157.6875	607436.75	1228.370728	6.749289712
154	676623.875	582552.5625	1190.508057	6.541253058
155	676338.375	582879.3125	1189.449097	6.535434597
156	656899.75	565172.625	1147.805908	6.306625869
157	612318.125	522983.5313	1057.697632	5.81152545
158	559163.9375	472901.4375	955.5456543	5.250250848
159	495824.2188	413459.375	833.4727173	4.579520425
160	414458.6563	336664.2813	685.6134644	3.767106947
161	293431.7188	220907.7344	481.7527466	2.646993113
162	146656.7813	79152.45313	247.1113586	1.357754718
163	143630.0469	78895.03125	253.9583435	1.395375514
164	262488.2813	193955.6406	445.3589783	2.447027353
165	383235.5938	309854.0313	645.9215698	3.549019614
166	464595.5625	386580.25	792.2054443	4.352777167
167	525097.0625	443339.7813	910.6968994	5.003829118
168	581379.875	495835.375	1018.299683	5.595053201
169	623179.4375	535737.0625	1105.15686	6.072290441
170	645929	556560.1875	1151.198608	6.325267079
171	646536.8125	556742.5	1152.345947	6.331571139
172	611850.8125	526658.8125	1109.939941	6.098571107
173	610818.0625	526114.125	1108.349731	6.089833689
174	591824.4375	508835.8438	1069.369263	5.87565529
175	554067.5	473203.0313	992.4127808	5.452817477
176	498555.4063	421064.9375	881.2527466	4.842048058
177	445371.0938	371361.3438	777.4182739	4.271528978
178	374926	304569.5938	646.788147	3.553781027
179	268889.0625	202923.0938	466.9551086	2.56568741
180	143688.3125	82077.90625	267.4565125	1.469541277
